# Molecular mechanism of bone metastasis in breast cancer

**DOI:** 10.3389/fonc.2024.1401113

**Published:** 2024-11-13

**Authors:** Laijian Sui, Jing Wang, Wen G. Jiang, Xicheng Song, Lin Ye

**Affiliations:** ^1^ Department of Orthopedics, Yantai Yuhuangding Hospital, Qingdao University, Yantai, Shandong, China; ^2^ Cardiff China Medical Research Collaborative, Division of Cancer and Genetics, Cardiff University School of Medicine, Cardiff, United Kingdom; ^3^ Department of Intensive Care Unit, Yantai Yuhuangding Hospital, Yantai, Shandong, China; ^4^ Department of Otorhinolaryngol and Neck Surgery, Yantai Yuhuangding Hospital, Qingdao University, Yantai, Shandong, China

**Keywords:** molecular mechanism, bone metastasis, breast cancer, bone microenvironment, relapse

## Abstract

Bone metastasis is a debilitating complication that frequently occurs in the advanced stages of breast cancer. However, the underlying molecular and cellular mechanisms of the bone metastasis remain unclear. Here, we elucidate how bone metastasis arises from tumor cells that detach from the primary lesions and infiltrate into the surrounding tissue, as well as how these cells disseminate to distant sites. Specifically, we elaborate how tumor cells preferentially grow within the bone micro-environment and interact with bone cells to facilitate bone destruction, characterized as osteoclastic bone metastasis, as well as new bone matrix deposition, characterized as osteoblastic bone metastasis. We also updated the current understanding of the molecular mechanisms underlying bone metastasis and reasons for relapse in breast cancer, and also opportunities of developing novel diagnostic approaches and treatment.

## 1 Introduction

Breast cancer is the leading malignant tumor in females that seriously threatens women’s health. In recent years, the number of patients suffering from this disease has been increasing year by year. In 2020, it has surpassed lung cancer for the first time and become the most frequently diagnosed cancer globally in the entire population, males and females combined (1). The global morbidity of breast cancer has been rising since the late 1970s. Although the mortality has decreased by 35% since the early 1970s owing to the progress in early diagnosis and advanced therapy, it is estimated that 10% to 12.5% of females may be attacked by this disease during their lifetime and the incidence is estimated to increase by 2% in the United Kingdom by 2035 (2).

Breast cancer metastasis is a multi-step complex process which originates from the local infiltration to the surrounding tissues by the primary breast cancer cells. The tumor cells detach from the primary tumor, infiltrate the surrounding tissues and penetrate into the blood or lymph vessels (3, 4). Then they spread to regional site and subsequent distant organs via lymphatic circulation and the blood circulation,respectively. Before settling down in the distant sites, disseminated breast cancer cells undergo cell cycle arrest and adhere to the capillary bed in the target organ. Once the number of the cell reach a certain amount, there is still no unified or definite standard, these dormant cancer cells may be woken up by the inflammation、stress hormones、estrogen deprivation、stromal injury, etc. to proliferate again (5). Meanwhile, tumor cells must escape from immune surveillance and apoptosis signals. After conquering these barriers, the tumor cells will achieve successful colonisation (4).

## 2 Molecular mechanism of distant dissemination of BC

### 2.1 Invasion

Invasion of breast cancer cells into surrounding tissues arises from alterations of intercellular adhesion and the adhesion between cells and the extracellular matrix (ECM). The role of the cadherin family is prominent in this process (6). E-Cadherin is vital in mediating cell-to-cell adhesion, and the downregulation of E-Cadherin leads to attenuated intracellular adhesion of breast cancer cells, leading to the detachment of cancer cells from the primary lesion (7). Therefore, downregulated E-cadherin is closely correlated with advanced invasion and poor prognosis of breast cancer (8). N-cadherin is closely associated with epithelial-mesenchymal transition (EMT) in breast cancer cells and proved to be another vital factor for tumor invasion (9). High expression of N-cadherin increases the adhesion of tumor cells to stromal cells which facilitates tumor cells to colonize the matrix (10). Cluster of differentiation(CD) 44 is also an important marker of EMT which exerts pivotal role in metastasis of breast cancer. Variant CD44 was detected from the standard isoform during EMT (11). Another EMT marker, α-smooth muscle actin (α-SMA), was highly expressed in carcinoma cells and associated with invasion of tumor invasion (12). EMT induces the production of proteases involved in ECM degradation to enhance the tumor invasion (13). ECM degradation enhances tissue penetration, which is also an essential step in tumor invasion. The degradation of ECM is mainly accomplished by Matrix Metallopeptidase (MMP) and urokinase plasminogen activator (uPA) (14). In breast cancer patients, uPA levels are closely related to the risk of distant metastasis (15). Inhibition of uPA by small interfering RNA (siRNA) can inhibit tumor invasion, and the expressions of MMPs are suppressed simultaneously (16). MMP-mediated degradation of ECM proteins is a prerequisite for breast cancer cell infiltration (17).

Increased expression of heparan sulphate proteoglycans (such as Glypican-1 and syndecan-1) has been observed in the advanced stages of breast cancer (18). Heparan sulphate proteoglycan is the proteoglycan in ECM or cell surface, which helps to maintain the integrity of ECM and mediate the interaction between cell matrix adhesion and growth factor receptor (19). Heparinase (a type of β-glucosidase) can promote ECM degradation by decomposing heparan sulphate proteoglycans (20). tumor cells can synthesize heparinase to degrade heparan sulphate to increase tumor cell invasiveness. Studies have confirmed that overexpression of heparinase in MCF7 cell-lines *in vitro* and *in vivo* promote cell proliferation and matrix invasion (21).

### 2.2 Migration and vitality

The migration of tumor cells can be accomplished individually or in a coordinated manner. Moderately and highly differentiated breast lobular carcinoma cells prefer to coordinate migration whilst poorly differentiated tumors are inclined to undergo single cell migration due to the abnormal structure and function of intercellular adhesion proteins (22). The co-migration of tumor cells requires a firm intracellular connection in case of being scattered. As a result, they usually aggregate as emboli after invasion in blood vessels (23). EMT is a key process in the mesenchymal movement of a single migrating cell. During EMT, tumor cells lose their epithelial phenotype (E-cadherin expression) and express mesenchymal markers, such as N-cadherin, SNAI1, SLUG (SNAI2), TWIST, vimentin, fibronectin (24). Breast tumor cells that undergo EMT are more aggressive. They can remodel their shapes to move through the degraded ECM with the least resistance (25). The transcriptional repressors of E-cadherin include E-box-binding homeobox 1 (ZEB1), zinc finger E-box-binding homeobox 2 (ZEB2), twist related protein (Twist), zinc Finger proteins, Snail and Slug, etc., which initiate EMT through TGF-β, Wnt, and phosphatidylinositol 3’kinase serine/threonine kinase (PI3K/AKT) pathway and indicate poor prognosis of breast cancer (26).

Tumor stromal cells promote tumor cell migration. Most stromal cells in breast cancer are fibroblasts, commonly referred to as cancer-associated fibroblasts (CAF). Conditioned medium collected from CAF can promote breast cancer cell motility and invasion *in vitro* (25).

### 2.3 Tumor microenvironment

The tumor microenvironment (TME) is composed of fibroblasts, immune cells, blood vessels and the extracellular matrix (ECM) (27, 28) and exerts crucial effect in tumor metastasis. The complexity of the TME means that tumor development and progression rely not only on the tumor cells themselves but also on stromal and immune cells. CAFs can provide both the physical support and direct the intracellular communications (29). CAFs undergo the Reverse Warburg effect and provide cancer cells with glycolytic metabolites (30), and CAF-derived exosomes can reprogram the metabolic pathway of cancer cells (31). ECM also provide the architectural support to faciliate the cell adhesion、water and growth factors preservation for the cancer cells. During the tumor progression, cancer cells lead to the stiffness of ECM, and the stiffen ECM contributes to abnormal proliferation、enhanced metastasis、immuno-suppression、resistance to theraputics in return (32). Macrophages in the tumor environment can interact with breast cancer cells and endothelial cells to form a niche to facilitate tumor colonization, proliferation, and escape from immune surveillance (33). T cells, neutrophils, and other immune cells also play crucial roles in breast cancer metastasis, influencing both the progression of the disease and the response to treatment. T cells are a critical component of the adaptive immune system and can infiltrate the TME where they exert anti-tumor effects. However, their function can be impaired within the TME due to various immunosuppressive mechanisms. Induced by IL1β, γδ T cells were capable of producing IL17, which leads to the systemic expansion and polarization of neutrophils dependent on G-CSF(Colony Stimulating Factor) in mice with mammary tumors. These tumor-generated neutrophils gain the capacity to suppress cytotoxic CD8+ T cells, which are crucial for controlling metastasis. Neutralizing IL17 or G-CSF, as well as the absence of γδ T cells, prevented the accumulation of neutrophils and reduced their T cell-suppressive characteristics. Furthermore, the lack of γδ T cells or neutrophils significantly diminished metastases in the lungs and lymph nodes without affecting the progression of the primary tumor. These findings suggest that targeting the newly identified immune pathway involving γδ T cells, IL17, and neutrophils could be a promising strategy to prevent metastatic disease (34). IL-22 induced by T cell helps to elevate CD155 expression by cancer cells, which interrupts NK cell function and activates immunosuppressive circuit to enhance lung metastasis (35). Increased CD8+ T cell infiltration with promoted T cell immunity infiltration was associated with reduced breast cancer distant recurrence (36). Activation of GM-CSF-JAK/STAT5-C/EBPβ pathway helps TINs to defense against ferroptosis via the Acod1-dependent immunometabolism, which inflicts antitumor T cell immunity and enhances metastasis (37). MHCII^hi^ neutrophils were shown to facilitate the metastasis of breast cancer to the lung, which were recruited by C-C Motif Chemokine Ligand 2 (CCL2) from lung tissues with chronic pulmonary infection, acts as the vital bacterial-immune mediator to bridge chronic infection and lung metastasis of breast cancer in a cell-intrinsic manner in a mouse model (38). Co-cultured BC cells with mesenchymal stem cells increased the expression of the receptor activator of nuclear factor κB (RANK) and epidermal growth factor receptor (EGFR) to facilitate osteoclastogenesis, which also indicated EGFR signalling could be a promising strategy to intercept bone metastasis (39). However, involvement of immunity and therapeutic opportunities in bone metastasis of breast cancer provoke more investment and intensive research.

Tumor cells themselves may affect the microenvironment of the secondary site before metastasis, establishing a “pre-metastasis niche” (40). Vascular endothelial growth factor receptor 1 (VEGFR-1)-positive clusters of hematopoietic progenitor cells are observed in the pre-metastatic lymph nodes of breast cancer patients before the tumor cells spread to the distant site (40). Chemokines are involved in the colonization of tumor cells to target organs. Chemokine receptor 4 (CXCR4) is highly expressed by breast cancer tissues, and its ligand, chemokine ligand 12 (CXCL12), is mainly in the lymph nodes. Organs with high CXCL12 expression are associated with some sites of metastatic breast cancer, such as the lung, bones and lymph nodes (41). The interaction of CXCR4-CXCL12 promotes the migration of breast cancer cells to the common site (41). Another important aspect of metastasis is neovascularisation, which provides nutrition and oxygen for metastases (42). Tumors grow faster than normal tissues and this easily leads to hypoxia in the lesions. Hypoxia stimulates production of pro-angiogenic factors in tumor cells and promote the formation of new blood vessels. For example, hypoxia-inducible factor-1 (HIF-1) triggers the production of an angiogenic protein vascular endothelial growth factor (VEGF) (42, 43). Through binding with specific VEGF receptors, VEGF can enhance the proliferation of vascular endothelial cells and increased the permeability of micro-vessels to induce neovascularisation (44). However, the new rapidly formed tumor vessels are excessively branched with varying shunts and diameters, which are different from the normal vessel both in structure and function. Abnormal blood vessels do not provide sufficient oxygen for the tumor, leading to a vicious cycle of tumor hypoxia (45). In breast cancer, the expression of VEGF indicates a poor prognosis and the tumor is prone to metastasis (46).

## 3 Bone metastasis of breast cancer

Bone metastasis is one of the most serious complications, which often occurs in the advanced stage of solid tumors such as lung, breast, prostate, colon-rectal, thyroid, gynecologic, and melanoma (47). Bone is one the most frequent sites for metastases and the morbidity of bone metastasis is about 70% in all metastatic breast cancer (48). As the advanced phase of breast cancer, bone metastasis is incurable and often leads to a debilitating disease with many other skeletal related events (SREs) including pathological fracture caused by osteolysis, dysfunction of the limb and bone marrow aplasia (49). Bone metastasis not only minimizes the life quality but also decreases the overall survival of the patients. Mortality in patients with bone metastases was significantly higher, especially for bone metastasis complicated by SREs (50).

### 3.1 Bone remodelling and the bone metastasis of breast cancer

Normal bone metabolism is the process within dynamic balance of bone remodeling which is well orchestrated by osteoblasts, osteoclasts and osteocytes. Bone remodeling is a continuous process where old bone is removed (bone resorption) and new bone is formed (bone formation). Bone remodeling, a process in which osteoclasts and osteoblasts coordinate with each other, can regulate calcium homeostasis, repair bone damage to resist stress and maintain skeletal system function. The remodeling process is initiated by various signals, including mechanical stress and hormonal changes (51). Osteoclasts are large, multinucleated cells responsible for the resorption of bone. Once the pre-osteoclasts are stimulated and differentiate into mature osteoclasts, osteoclasts attach to the bone matrix and form resorption lacunae by secreting enzymes and acids to dissolve the mineral matrix and collagen fibers.This process releases minerals like calcium and phosphate into the bloodstream (52). After resorption, the area undergoes a transitional phase where the resorbed bone surface is prepared for new bone formation. Osteoblasts, known as mesenchymal stem cells differentiate from precursor cells, are responsible for the formation of new bone. They produce new bone matrix and initiate its mineralization (53). Osteocytes are mature bone cells that originate from osteoblasts. They become embedded in the bone matrix and help maintain the bone tissue. Osteocytes communicate with other bone cells to regulate the remodeling process (54). Studies revealed that several molecular mechanisms promote the bone resorption process, in which the receptor activator of nuclear factor κB (RANK) and its ligand (RANKL) are critical in regulating osteoclast function (55). Osteoclast precursors (monocytes/macrophages lineage) express a receptor called RANK (Receptor Activator of Nuclear factor Kappa-B). RANKL binds to RANK on the surface of these osteoclast precursors The binding of RANKL to RANK initiates a cascade of intracellular signaling events within the osteoclast precursor, primarily involving the activation of the NF-κB (Nuclear Factor Kappa-B) pathway and other downstream signaling pathways like MAPK (Mitogen-Activated Protein Kinase) (55, 56). These signaling pathways promote the differentiation and maturation of osteoclast precursors into fully functional multinucleated osteoclasts. Mature osteoclasts attach to the bone matrix and secrete hydrochloric acid (HCl) and lysosomal enzymes, like cathepsin K, which degrade the organic matrix and dissolve the mineral components of bone (57).This activity creates small pits or resorption lacunae on the bone surface, effectively re-absorbing bone tissue. Osteoblasts and other cells also produce OPG (osteoprotegerin), a decoy receptor that binds to RANKL, preventing it from interacting with RANK. This serves as a natural inhibitor of RANKL-mediated osteoclastogenesis and bone resorption (58). Besides RANKL pathway, hormones, cytokines and growth factors influence the proliferation of osteoclasts and osteoblast progenitor cells. Parathyroid hormone (PTH) induced the generation of osteoclast to enhance osteoclast mediated bone resorption (59). IL-6, IL-8, TGF-β, and other molecules are also involved in stimulating osteoclast activity and supporting cancer cell survival and growth in bone (**Table 1**). Sex steroids inhibited maturation of osteoclasts, and reduced secretion of sex hormone enhanced the activity of osteoclasts, which may lead to apoptosis of bone cells to promote bone resorption (60).

**Table 1:** Signalling pathways associated with the bone metastasis in breast cancer.

Cytokines/Pathways	Roles
TGF-β	TGF-β is released by cancer cells and bone matrix during bone resorption. It promotes the epithelial-mesenchymal transition (EMT) in cancer cells, enhancing their migratory and invasive capabilities (123).
BMPs	BMPs are involved in bone formation and are also implicated in promoting the survival and growth of metastatic cancer cells in the bone (124, 125).
ILs	IL-6: Supports cancer cell proliferation and survival; also promotes osteoclast differentiation, which leads to bone resorption (126). IL-8: Enhances the invasiveness and migration of breast cancer cells (127). IL-11: Contributes to osteoclastogenesis and bone resorption (128).
PTHrP	PTHrP is secreted by breast cancer cells and stimulates osteoclast activity indirectly by enhancing RANKL expression, leading to bone resorption (59).
M-CSF	Supports the formation and survival of osteoclasts, contributing to bone resorption (129).
Prostaglandin E2	Promotes the maturation and activity of osteoclasts via inducing RANKL expression ro facilitate bone resorption (130).
OPN/BSP	Induces the proliferation of osteoblast, to promotes bone resorption (131).
RANK/RANKL/OPG Pathway:	RANK is osteoclast precursors (132). RANKL is expressed by osteoblasts and stromal cells, which binds to RANK to promote osteoclast differentiation and activation (67, 68) (133). OPG: A decoy receptor for RANKL, inhibiting its interaction with RANK and thus decreasing osteoclastogenesis (132).
Integrins and Focal Adhesion Kinase (FAK) Pathway:	Integrins facilitate the attachment of cancer cells to the bone matrix (134). FAK signaling promotes cell survival, proliferation, and migration (134).
PI3K/AKT/mTOR Pathway:	Key pathway for cell survival and proliferation. Activation leads to cancer cell growth and resistance to apoptosis (135).
Wnt/β-catenin Pathway:	Wnt signaling is involved in bone formation and cancer progression (136), whilst β-catenin accumulation can lead to increased cancer cell proliferation and survival (136).
MCP-1/CCR2 Pathway:	Monocyte chemoattractant protein-1 (MCP-1) and its receptor CCR2 are important for recruiting monocytes/macrophages, which can differentiate into osteoclasts and promote bone resorption (137).
VEGF:	VEGF enhances angiogenesis, providing nutrients and oxygen to metastatic cancer cells, and is also implicated in promoting osteoclastogenesis and bone resorption (138).

The process of bone metastasis of breast cancer is a multiple-step cascade which contains four main steps: (1) invasion, proliferation and dissociation of cancer cells from the primary lesion, (2) intravasation and dissemination in the circulation (3) extravasation of cancer cells (4) colonization in the bone, disseminated tumor cells settle down in the bone niche, where it is normally hosted by hematopoietic stem cells (HSCs), followed by a survival under dormancy, reactivation and ultimate outgrowth (61, 62).

The bone matrix itself provides a physical barrier which may impede the colonization of cancer cells. Bone marrow is an immunological niche where immune cells like T-cells and natural killer (NK) cells can survey and eliminate aberrant cells (63), including cancer cells. Osteoblasts and other bone marrow stromal cells can out-compete cancer cells for space and nutrients, limiting their growth. The bone matrix releases anti-angiogenic factors including angiostatin, interferons (α, β and γ), endostatin, interleukin-12 and retinoids that can suppress the formation of new blood vessels that are required during tumor growth (64). However, once the dynamic balance is destroyed, it may lead to osteolytic lesions, presenting lower bone density or osteoblastic lesions, with excessive bone deposition. After menopause, due to the rapid decline in oestrogen levels, osteoclasts are active and bone loss is accelerated. After breast cancer patients received chemotherapy or hormone adjuvant therapy, the risk of low bone density and osteoporosis has been found to be increased (65). When breast cancer cells spread to the bones, they will gradually adapt to the bone microenvironment, destroy the bone homeostasis, then, start a vicious cycle of bone metastasis under various mechanisms.

### 3.2 Predilection to metastasis to the bone

Although it is lacking in understanding, characteristics of bone environment and properties of breast cancer cells certainly bear traits at levels of tissues, cells and genes for the predisposition of bone metastasis from breast cancer. The inorganic phase of bone is mainly composed of the mineral hydroxyapatite nanocrystals (HA). High HA induced the secretion of pro-osteoclastic interleukin-8 (IL-8) by MDA-MB-231 cells to facilitate bone colonization (66). The extracellular bone matrix is enriched with type-I collagen, osteopontin (OPN), and bone sialoprotein (BSP). Elevated expression of OPN and BSP can facilitate tumor cell adhesion to collagen and increase metastatic propensity to bone (67, 68). The skeletal microenvironment is known to be a highly hypoxic environment and the pressure of oxygen (pO2) in mouse bone marrow is significantly lower than other tissues or organs (69). Hypoxia is known to be involved in various steps of bone metastasis, including the premetastatic niches, dormancy and osteolytic vicious cycles (70, 71). Bone marrow hypoxia can promote the expression of HIF-1 (72), which subsequently induce the secretion of C-X-C motif chemokine 12 (CXCL12) (73). Upon binding with Ca^2+^ and chemokine receptor 4 (CXCR-4), CXCL12 activate multiple signalling pathways such as PI3K/Akt, ERK/MAPK pathway to facilitate the colonisation of disseminated tumor cells in bone tissue (74). Hypoxia could also enhance the activity of osteoclasts and suppress the differentiation of osteoblasts (70). The bone environment contains a lot of alkaline minerals (hydroxyapatite) and the buffer system to maintain a normal pH value. In the early stage of bone metastasis, due to the hypoxia and excess secretion of H+ both inside and outside of the cell membrane, caused by a high glycolysis status in the tumor cells, the bone microenvironment is maintained in a state of acidosis. Among this process, vacuolar H^+^-ATPase (V-ATPase) performed actively for bone microenvironment acidosis, which was expressed in both tumor cells and osteoclasts (75). Acidosis significantly enhanced the activity of osteoclasts with elevated secretion of cytokines, leading to bone loss, such as activated T-cell nuclear factor 1 in activated osteoclasts (76). Acidosis could also inhibit the biological functions of osteoblasts, leading to impaired trabecular bone formation and promoted the expression of osteoclast RANKL (77). In addition, the acidosis environment activated NF-κB signal transduction pathway in mesenchymal stromal cell can promote the secretion of inflammatory factors, chemokines and growth factors, such as IL-1, IL-6 and CXCL2, which can subsequently induce tumor-induced nociception and hyperalgesia to facilitate invasion and immune escape (75).

Trabecular of cancellous bone is fenestrated which contains rich blood vessels with slow blood flow and is suitable for breast cancer cells to colonise after successfully spreading through blood circulation (78).

Various kinds of tumor cells including ovarian, gastric and colorectal cancers can be detected in the bone marrow (79–81), which indicates that the bone metastasis of breast cancer at the initial stage is passive. However, only a few kinds of cancer cells including breast cancer cells can form overt metastatic bone lesions (49), which indicates that passive dissemination of breast tumor cells to the bone marrow is an early step in forming bone metastasis, but it is not the critical driving event of bone metastasis. Apart from the passive transportation, the properties of breast cancer cells are essential in bone metastasis. The bone environment is a reservoir for minerals, especially for calcium ions. Breast cancer cells highly express calcium-sensing receptor (CaSR), which could bind with Ca^2+^, and promote breast tumor cell spread to the bone tissue with high Ca^2+^ concentration. *In vitro* studies have shown that extracellular Ca^2+^ combined with CaSR expressed by tumor cells activate AKT and MAPK pathways to enhance migration and proliferation of cancer cells, whilst application of CaSR antagonists to interfere with renal cancer in mice significantly reduced the incidence of bone metastasis (82).

**Figure 1:**
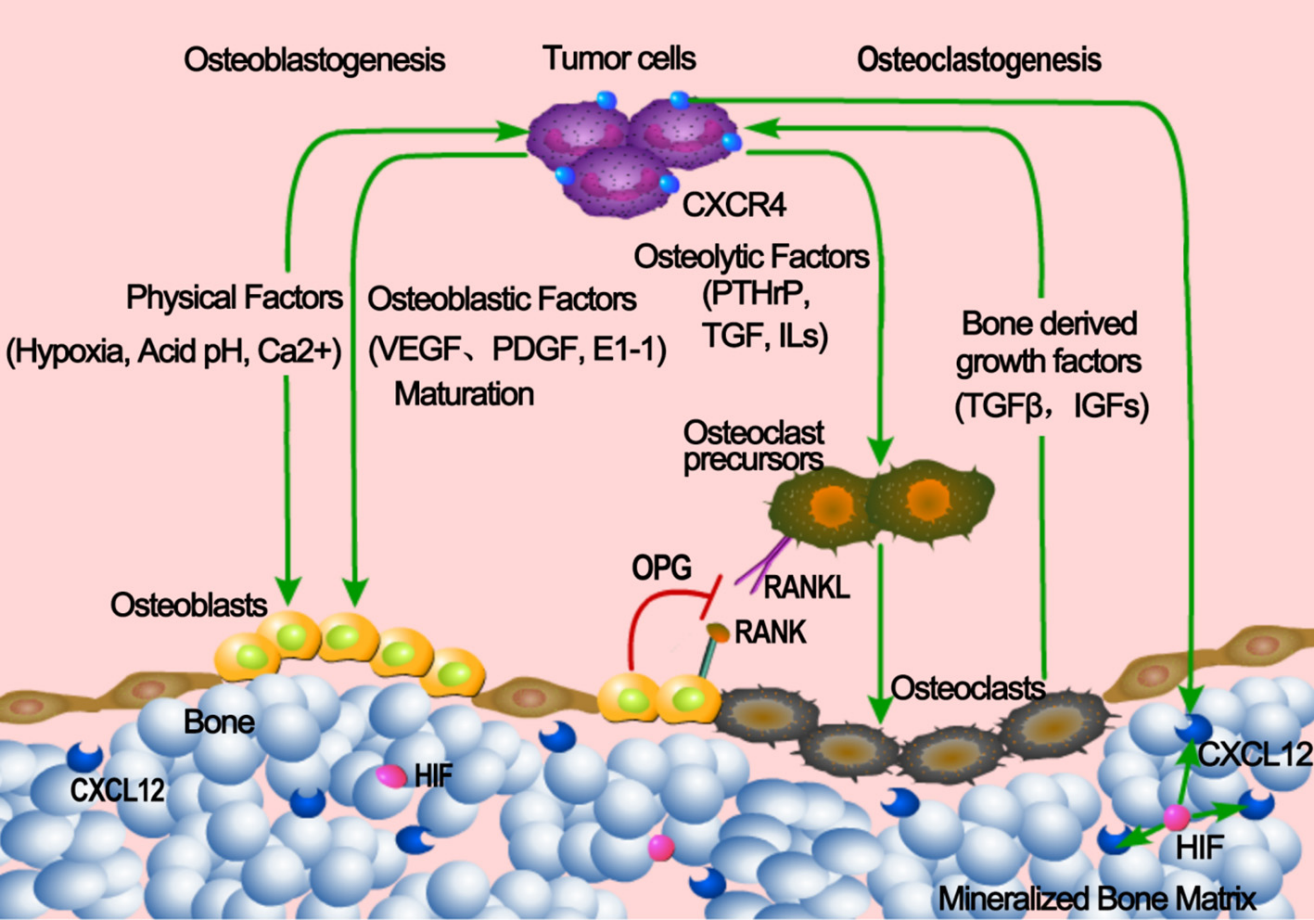
Mechanism of bone metastasis in breast cancer. Osteolytic factors such as PTHrP, TGFβ, IL11, IL6 etc. secreted from the tumor cells induce the maturation of osteoclast from precusers, leading to the bone destruction. tumor cells also secrete osteoblastic factors including VEGF, PDGF etc. to promote the maturation of osteoblast to induce bone formation. CXCR4 was highly expressed in breast cancer tissues, and its ligand CXCL12 was overexpressed in bone marrow induced by HIF. RANKL produced by the osteoblast also contributes to the maturation of osteoclast to lyse the bone matrix. Released growth factors due to the bone destruction support the expansion of tumor cells in turn and aggravate the bone destruction. This process is named vicious cycle.

RANK was highly expressed on the surface of breast cancer cells, while RANKL was overexpressed in bone tissues (83, 84). In addition, the chemokine receptor CXCR4 was highly expressed in breast cancer tissues, and its ligand CXCL12 was overexpressed in common metastatic sites of breast cancer including bone marrow (41). Interaction mediated by these molecules between cancer cells and bone microenvironment may account for at least partially for the predisposition of breast cancer to metastasize to the bone (**Figure 1**).

### 3.3 Biological character of bone metastasis in breast cancer

Bone metastasis is preferential in poorly differentiated and ER-positive breast tumors (85), upregulated trefoil factor-1 (pS2 or TFF1) was reported as the potential biomarker for the predilection of bone metastasis in early stage of ER-positive breast carcinoma rather than the advanced stage (86). Notably, although the early incidence of bone metastasis in ER-negative cancers is higher, the frequency of bone recurrence is higher in ER-positive cancers (87), which may caused by the reduced ER expression and activities by osteogenic niche in bone micrometastases, leading to the endocrine resistance (88). Lymph circulation plays a vital role in the invasion of breast cancer and the lymph node status is very important in predicting the prognosis of breast cancers. Generally, the assessment of the risk of developing bone metastasis in breast cancer includes the number of positive lymph nodes, the size of the tumor, and age of the patient. If the numbers of positive lymph nodes are more than 4, and the size of the tumor is larger than 2cm and the patient is younger than 35 years, the patients are usually associated with higher risk of bone metastasis (87).

#### 3.3.1 Dormancy

After disseminating and settling in the bone, breast cancer cells often enter a quiescent state to evade host immunosurveillance and adapt to the bone environment. This state is characterized by cell cycle arrest at the G0/G1 phase, thus lacking obvious proliferative features (89, 90). The duration of dormancy is subtype-dependent. In luminal A/B breast cancers, dormancy can last up to 10 years, maintaining a steady probability of metastatic relapse. Conversely, in triple-negative breast cancer (TNBC), bone metastases are typically developed within 5 years following diagnosis (91).

The hypoxic environment of the primary tumor influences the dormancy of metastatic cells in bones. Hypoxia in primary tumors induces a dormant gene program mediated by NR2F1 (Nuclear Receptor Subfamily 2 Group F Member 1), leading to dormancy in the bone microenvironment (92). Bone marrow, the site of hematopoietic stem cell (HSC) production, consists of the perivascular and endosteal niches. Both niches involve cells such as endothelial cells and osteoblast lineage cells that secrete growth and apoptosis signals to maintain the HSC population (93). Tumor cells interacts with different niche cells to achieve bone colonization. Chemokines like CXCL12 and E-selectin are abundantly present in the bone microenvironment, attracting breast cancer cells to the perivascular niche to faciltate mesenchymal-to-epithelial transition(MET), stemness and survival (94). CXCL12 interacts with CXCR4, a receptor expressed on breast cancer cells, to guide their migration toward high-CXCL12 areas. After MET, distant breast cancer cells present a more static and epithelial state with to facilitate the subsequent metastatic outgrowth (95). E-selectin, an adhesion molecule on endothelial cells, facilitates initial tethering and rolling of cancer cells to active the Wnt signaling pathway—a crucial process for metastasis in distant organs (94, 96). It is worth mentioning that although epithelial markers such as EpCam and Keratin-14 increased in cancer cells, traditional EMT regulators (Snail, Twist and Zeb) remained unchanged after MET (97). Moreover, endothelial cells in the bone microenvironment can secrete angiogenesis inhibitors such as thrombospondin-1 (TSP1), which promote dormancy in disseminated breast cancer cells (98). The collaboration between the endosteal niche and the HSC niche provides a supportive microenvironment for metastatic cancer cell dormancy (99, 100). In the endosteal niche, tumor cells are also capable of expressing the Notch ligand Jagged1、vascular cell adhesion molecule (VCAM)1 or by inducing osteogenic cells to produce the osteoclast-stimulating factors macrophage colony stimulating factor (M-CSF) and RANKL to inducing osteoclastic bone formation, which facilitates the renowned ‘vicious cycle’ of osteolytic bone metastasis (94).

Internal tumor signaling also influences metastatic dormancy. The p38 MAPK pathway is instrumental in regulating tumor dormancy. Bone morphogenetic proteins (BMPs) in the bone microenvironment upregulate p38 and downregulate ERK expression, thereby inducing dormancy (101). The TGF-β2 cytokine, enriched in bone marrow, also contributes to inducing dormancy (90). Mitogen- and stress-activated kinase 1 (MSK1), a downstream effector of p38 MAPK, modulates breast cancer dormancy by altering chromatin structure and reducing luminal differentiation gene expression (e.g., GATA3, FOXA1) (102). Downregulation of MSK1 enhances the proliferation of bone-disseminated breast cancers, correlating with advanced metastasis in patients (102, 103). The orphan nuclear receptor NR2F1, another mediator of p38 MAPK, can initiate dormancy in several cancer types, including breast cancer (104). Clinical evidence associates NR2F1 expression with early breast cancer recurrence (105). Moreover, autophagy, independent of Beclin 1 (BECN1), is a survival mechanism inducing dormancy in breast cancer cells (106).

#### 3.3.2 Outgrowth

Upon adapting to the bone microenvironment, the balance between proliferation and apoptosis in breast cancer cells is disrupted, initiating a positive feedback loop involving tumor cells, osteoclasts, osteoblasts, and the bone matrix, termed “the vicious cycle of bone metastasis” (107). Disseminated breast cancer cells can undergo EMT to acquire an osteoblast-like phenotype, a process known as osteomimicry, characterized by the upregulation of pro-osteoblastic genes (108). Osteomimicry enables these cancer cells to functionally mimic osteoblasts and act as paracrine regulators of osteoclasts. Cytokines such as the receptor activator of nuclear factor-κB (RANK), interleukin-1 (IL-1), IL-6, IL-11, macrophage inflammatory protein 1a (MIP1a), M-CSF, and parathyroid hormone-related peptide (PTHrP) are secreted by osteo-mimicking tumor cells to enhance osteoclast formation and activity, leading to excessive bone resorption (109). These cells also upregulate RANKL expression on osteoblasts, stimulating osteoclast activity (110). Osteoblasts secrete osteoprotegerin (OPG), a decoy receptor that inhibits RANKL-induced osteoclastogenesis by competitively binding RANKL (111). PTHrP, released by osteoblasts, is a key regulator of the vicious cycle, enhancing osteoclastogenesis via RANKL and inhibiting OPG (112, 113). The resulting bone resorption provides new niches for cancer cell colonization and releases cytokines like BMP, transforming growth factor-β (TGF-β), fibroblast growth factor (FGF), and platelet-derived growth factor (PDGF) from the bone matrix, further promoting tumor proliferation and PTHrP production, thus perpetuating bone destruction (109). The subsequent calcium release during osteolysis can induce hypercalcemia. Breast cancer cells express calcium-sensing receptors that interact with released calcium ions to promote cell proliferation and survival (114). The Wnt signaling pathway also plays a role in enhancing osteoclast differentiation while inhibiting osteoblast activity (115). Dickkopf-1 (DKK-1), highly expressed in breast cancer patients with bone metastasis, inhibits Wnt signaling, promoting osteoclastogenesis and suppressing osteoblast function (116).

Breast cancer bone metastases predominantly exhibit osteolytic lesions, although osteogenic (osteoblastic) lesions are seen in about 12-50% of cases (117). Bone destruction in osteolytic lesions can induce reactive osteogenesis, leading to mixed lesions (109). The molecular mechanisms underlying osteoblastic lesions remain underexplored, yet Cbfα1 (Runx-2) is associated with osteoblastic differentiation and essential metastatic processes (118). Bone resorption biomarkers such as NTX (N-telopeptide of type I collagen) are elevated in osteoblastic disease, with the NTX/creatinine ratio used to monitor bone resorption (119). Osteoblast cadherin (CDH11) is another stromal interaction protein linked to osteoblastic metastasis (120). PDGF, FGF, TGF-β, BMP, and endothelin-1 are cytokines enhancing osteoblast activity (121). Endothelin-1 inhibits DKK-1 expression in bone marrow stromal cells, promoting osteoblast production and osteoblastic lesion development when Wnt signaling inhibition is alleviated (122). Signalling pathways associated with the bone metastasis in breast cancer were summarised in **Table 1**.

## 4 Conclusions and perspectives

In summary, the occurrence of bone metastasis is related to the biological behavior of tumor cells and the bone microenvironment. During bone metastasis, tumor cells and osteoblasts remotely regulate each other, and the bone microenvironment undergoes significant changes at the cellular and cytokine levels. However, further research is required to unveil the relationship between osteoclasts, osteoblasts and tumor cells, as well as the involvement of cytokines. It is believed that with the improvement of molecular and genetic technology, the molecular mechanism of bone metastasis will be clarified gradually, which will shed light on a theoretical basis for the earlier diagnosis and exploration of and novel anti-bone metastasis drugs.
